# Heparin-like effect in postcardiotomy extracorporeal membrane oxygenation patients

**DOI:** 10.1186/s13054-014-0504-2

**Published:** 2014-09-05

**Authors:** Marco Ranucci, Ekaterina Baryshnikova, Giuseppe Isgrò, Concetta Carlucci, Mauro Cotza, Giovanni Carboni, Andrea Ballotta

**Affiliations:** Department of Cardiothoracic and Vascular Anesthesia and Intensive Care, IRCCS Policlinico San Donato, Via Morandi 30, San Donato Milanese, Milan, 20097 Italy

## Abstract

**Introduction:**

Unfractionated heparin (UFH) is the anticoagulant of choice for extracorporeal membrane oxygenation (ECMO), but bivalirudin can be used as an alternative. The purpose of the present study is to investigate the existence of a heparin-like effect (HLE) during heparin-free ECMO.

**Methods:**

This is a retrospective study on patients treated with ECMO and receiving bivalirudin as the sole anticoagulant. Thromboelastography (TEG) tests with and without heparinase were recorded during the ECMO duration. A total of 41 patients (22 pediatrics and 19 adults) treated with ECMO after cardiac surgery procedures and receiving only bivalirudin-based anticoagulation were studied. Based on the presence of a different reaction time (R-time) between the TEG test with heparinase or without heparinase we defined the presence of a HLE. Survival to hospital discharge, liver failure, sepsis, bleeding and transfusion rate were analyzed for association with HLE with univariate tests.

**Results:**

HLE was detected in 56.1% of the patients. R-times were significantly shorter in tests done with heparinase versus without heparinase during the first seven days on ECMO. Patients with HLE had a significantly (*P* = 0.046) higher rate of sepsis (30%) than patients without HLE (5.6%) at a Pearson’s chi-square test.

**Conclusions:**

A heparin-like effect is common during ECMO, and most likely due to a release of heparinoids from the glycocalyx and the mast cells, as a consequence of sepsis or of the systemic inflammatory reaction triggered by the contact of blood with foreign surfaces.

## Introduction

Patients under extracorporeal membrane oxygenation (ECMO) require anticoagulation to prevent circuit and intravascular thrombosis. The usual anticoagulant is intravenous unfractionated heparin (UFH), at variable doses ranging from 20 IU/kg/h to 70 IU/kg/h [1]; however, alternative anticoagulants like bivalirudin have been proposed in patients with [2,3] or without heparin-induced thrombocytopenia [4–6].

At our institution, bivalirudin is the first choice for post-cardiotomy veno-arterial or veno-venous ECMO both in adult and pediatric patients, unless there is evidence of blood stagnation inside the cardiac chambers [5,6]. The assessment of the adequacy of anticoagulation includes serial measurements of the activated clotting time (ACT), activated partial thromboplastin time (aPTT), and thromboelastography (TEG).

**Figure 1:**
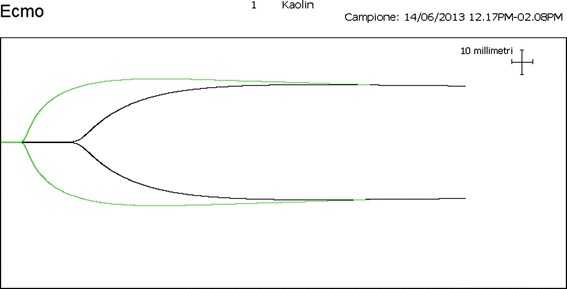
**An example of heparin-like effect on thromboelastography (kaolin activation).** Black tracing, test without heparinase; green tracing, test with heparinase. ECMO, extracorporeal membrane oxygenation.

By running TEG examination with and without heparinase, we started noticing different patterns of TEG profiles in some patients. One typical pattern observed in these cases is represented in Figure 1, and is characterized by a remarkably shorter reaction time (R-time) when the test is carried out with kaolin activation plus heparinase with respect to kaolin activation alone. This is suggestive for a heparin-like effect (HLE), in absence of any UFH treatment.

The present study is a retrospective review of a series of patients treated with heparin-free ECMO. The aim of the study is to confirm our hypothesis that ECMO patients may experience a heparin-like effect during the course of the ECMO treatment.

## Methods

Patients undergoing ECMO treatment at our institution are routinely included in an ECMO registry. The first patient treated with bivalirudin as the sole anticoagulant was reported in March 2010. From March 2010 to February 2014, 68 ECMO procedures were recorded. Twenty-seven patients were treated with UFH on ECMO. Forty-one patients were treated with bivalirudin as the sole anticoagulant during ECMO. For these patients, we have retrieved the TEG data measured during the ECMO period, which is stored in the memory file of the TEG device, the values of aPTT and international normalized ratio (INR), and transfusion data (packed red cells, fresh frozen plasma, and platelet concentrates).

The present study was approved by the local ethics committee (San Raffaele Hospital ethics committee), and the need for informed consent from the patients was waived, given the retrospective nature of the study and the existence of all data in a dedicated registry. At hospital admission, all patients gave written approval for the treatment of their data in an anonymous form for scientific purposes.

### Patient population

Forty-one patients suitable for the present analysis were identified. Patients who received heparin after the ECMO placement were excluded from the analysis. A control group of 12 patients not on ECMO was included; these patients were receiving TEG analysis for diagnosis of bleeding on the first day after cardiac surgery.

### ECMO procedure

The following ECMO circuits have been used during the study period: for patients below 10 kg body weight the circuit was assembled using a centrifugal pump Biomedicus BP50 (Medtronic, Minneapolis, MN, USA) and an oxygenator Dideco Lilliput 2 ECMO (Sorin Group, Mirandola, Italy) with phosphorylcholine-biocompatible treatment. For patients weighing >10 kg different circuits have been used according to availability: the centrifugal pumps used were the Biomedicus BP 80 (Medtronic), the Stockert Revolution (Sorin Group), the Levitronix (Waltham, MA, USA) equipped with the Dideco EOS ECMO (Sorin Group) oxygenator with phosphorylcholine-biocompatible treatment (Phisio, Sorin Group). Starting in 2012, a pre-assembled ECMO system (Permanent Life Support, Maquet, Rastatt, Germany) with a Rotaflow centrifugal pump, and a Quadrox PLS oxygenator with Bioline® coating was used in adult patients. The ECMO was usually performed at full flow, under normothermic conditions guaranteed by the oxygenator heat exchanger, a heater-cooler external group, and external heat-exchanging blankets.

### Anticoagulation protocol and transfusion policy

Anticoagulation monitoring during ECMO was obtained using the ACT, the aPTT, and kaolin-activated thromboelastography (TEG, Haemoscope, Niles, IL, USA). The ACT was initially repeated every 4 h and the target value was 160 to 180 seconds. Subsequently, it was progressively replaced by the TEG, repeated every 8 h, with a target value for the R-time placed at a minimum of 12 and a maximum of 30 minutes. The aPTT was repeated every 12 h, with a target value of 50 to 80 seconds.

The initial anticoagulation protocol was different depending on the timing of the ECMO implant. In patients under cardiopulmonary bypass with full heparin treatment, where the ECMO was implanted due to the inability to wean the patient from the cardiopulmonary bypass, the heparin was fully antagonized with protamine after the ECMO implantation, and the effectiveness of heparin antagonization was checked with ACT. Subsequently, the patient was observed for bleeding control. In the case of active bleeding (>4 mL/kg/h) no anticoagulants were used. Once the blood loss was contained and stabilized, the anticoagulation protocol was started and modulated to stick to the optimal coagulation values previously described.

In patients where the systemic heparin used for the cardiac operation was already antagonized, and the ECMO was implanted subsequently to protamine administration, a bolus dose of heparin (100 IU/kg) was used for cannulation. Once the ECMO was established, the heparin was fully antagonized with protamine and again the patient entered the observation period for severe bleeding before starting any anticoagulation treatment.

Once major bleeding was ruled out, all patients started anticoagulation with a continuous infusion of bivalirudin at an initial dose of 0.05 μg/kg/h. The dose was subsequently adjusted according to the ACT, aPTT, and R-time values. In patients with reduced creatinine clearance, the starting dose was halved. Details of the bivalirudin-based anticoagulation protocol have been previously reported [6].

### Data collection and definitions

For each patient we retrieved the demographic data, the nature of the ECMO treatment (veno-venous or veno-arterial), the use of phosphorylcholine or heparin coating of the circuit and oxygenator; and some outcome indicators including peak serum bilirubin value during ECMO, sepsis (signs of systemic inflammatory reaction and positive blood cultures), bleeding (mL/kg/day), and survival to hospital discharge. Additionally, we retrieved all the TEG tracings collected during the ECMO period. We recorded the R-time of kaolin-activated whole blood with or without heparinase. For every day on ECMO, we recorded the R-times having the larger difference between TEG tracings with or without heparinase. Therefore, we obtained the daily values of R-times more representative of the phenomenon we wanted to explore. The observation period was extended to no more than 12 days on ECMO. For the purposes of the present study, we defined an HLE as the presence of at least one TEG analysis showing an R-time of the test with heparinase at least 30% shorter than the correspondent R-time of the test without heparinase.

### Statistics

Normality of continuous data distribution was checked with a Kolgomorov-Smirnov test. Differences between R-times with or without heparinase were tested with the paired data Student’s *t*-test. Time-related differences were investigated with analysis of variance for repeated measures. Associations between HLE and other parameters were explored using Pearson’s chi-square test, Student’s *t*-test, or non-parametric tests when appropriate. A *P*-value <0.05 was considered significant for all the tests applied. For all the statistical analyses a computerized package (IBM SPSS 20.0, Chicago, IL, USA) was used.

## Results

The characteristics of the patient population are shown in Table 1. The 22 pediatric patients included 7 newborn babies (weight 3.2 ± 0.6 kg, range 2.3 to 4.0 kg), 9 infants age ≤1 year weight 6.0 ± 1.2 kg, range 4.6 to 7.6 kg) and 6 patients age >1 year (weight 26.1 ± 15.0 kg, range 13 to 45 kg). They were submitted to different corrective or palliative procedures, including six arterial switch operations for transposition of the great vessels, six valvular repair/replacements, two systemic-to-pulmonary shunts, one complete atrioventricular canal, one Rastelli operation, one total anomalous pulmonary venous return, one Norwood operation and four complex, combined procedures.

**Table 1:** **Characteristics of the patient population, of the ECMO, and outcome data**

**Variable**	**Overall (n = 41)**	**HLE (n = 23)**	**No HLE (n = 18)**	***P*** **-value**
Pediatric patients (<16 years), number (%)	22 (53.7)	12 (52.2)	10 (55.6)	0.829
Veno-venous ECMO, number (%)	8 (19.5)	5 (21.7)	3 (16.7)	0.684
Veno-arterial ECMO, number (%)	33 (80.5)	18 (78.3)	15 (83.3)	
Phosphorylcholine coating, number (%)	29 (70.7)	15 (65.2)	14 (77.8)	0.380
Heparin coating, number (%)	12 (29.3)	9 (34.8)	4 (22.2)	
Days on ECMO, median (range)	6 (1 to 12)	7 (3 to 12)	4 (1 to 12)	0.008
Bleeding on ECMO (mL/kg/day), mean (SD)	17 (13)	15.5 (14)	20.6 (11)	0.144
Peak serum bilirubin on ECMO, mg/dL, mean (SD)	4.4 (5.7)	3.7 (3.2)	5.3 (7.6)	0.410
Sepsis, number (%)	8 (19.5)	7 (30.4)	1 (5.6)	0.046
Survived to hospital discharge, number (%)	19 (46.3)	9 (39.1)	10 (55.6)	0.295

ECMO, extracorporeal membrane oxygenation; HLE, heparin-like effect.

There were 23 (56.1%) patients fulfilling the criteria for HLE. We could not identify any factor related to age and ECMO characteristics showing an association with HLE. Patients who demonstrated an HLE had a significantly (*P* = 0.008) longer duration of ECMO. There was no association between HLE, bleeding, and peak serum bilirubin values. Conversely, there was a significantly (*P* = 0.046) higher rate of patients with sepsis in the HLE group.

**Figure 2:**
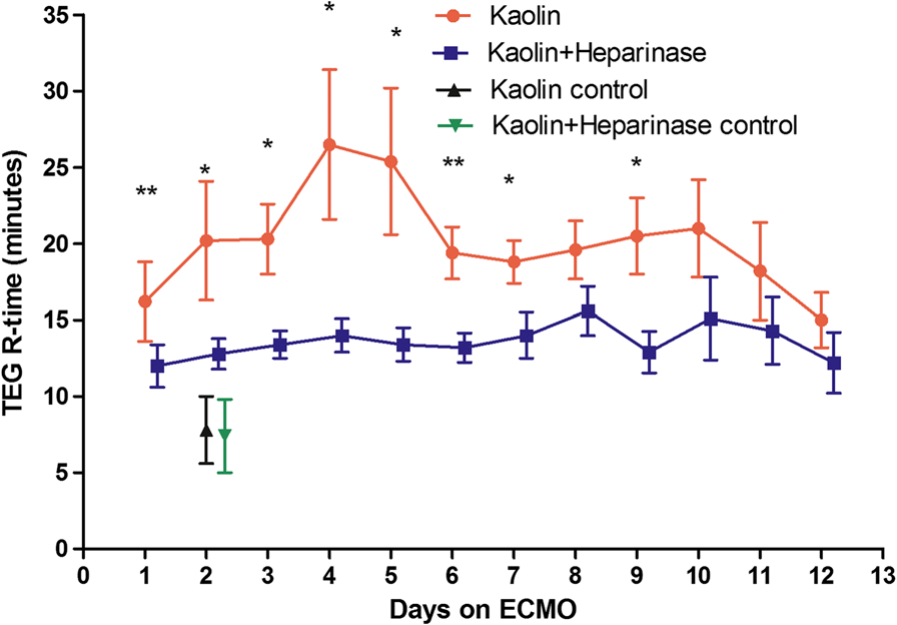
**Reaction times (R-time) on thromboelastography (TEG) with and without heparinase during the first 12 days on extracorporeal membrane oxygenation (ECMO).** Data refer to the whole patient population and are mean values of the different tests done on the same day. *P <0.05; **P <0.01.

The patterns of the R-times with and without heparinase are shown in Figure 2. At any point in time, the R-time of the TEG with heparinase was shorter than the correspondent R-time of the TEG without heparinase. This difference was statistically significant on day 1 through day 7 (with *P*-values ranging from 0.004 to 0.047) and on day 9 (*P* = 0.012). On day 8, and from day 10 through day 12, no significant difference was observed. The overall effect of time was not significantly associated with the changes in R-time at the TEG with (*P* = 0.909) or without (*P* = 0.652) heparinase.

The first TEG done immediately after positioning of the ECMO and protamine antagonization of heparin demonstrated a small, non-significant difference in R-times, with an R-time of 10.7 ± 4.5 minutes for TEG without heparinise and 9.6 ± 4.0 minutes for TEG with heparinase. In the control group of patients analyzed with TEG with or without heparinase, there was no significant difference in the R-time 24 h after surgery.

**Figure 3:**
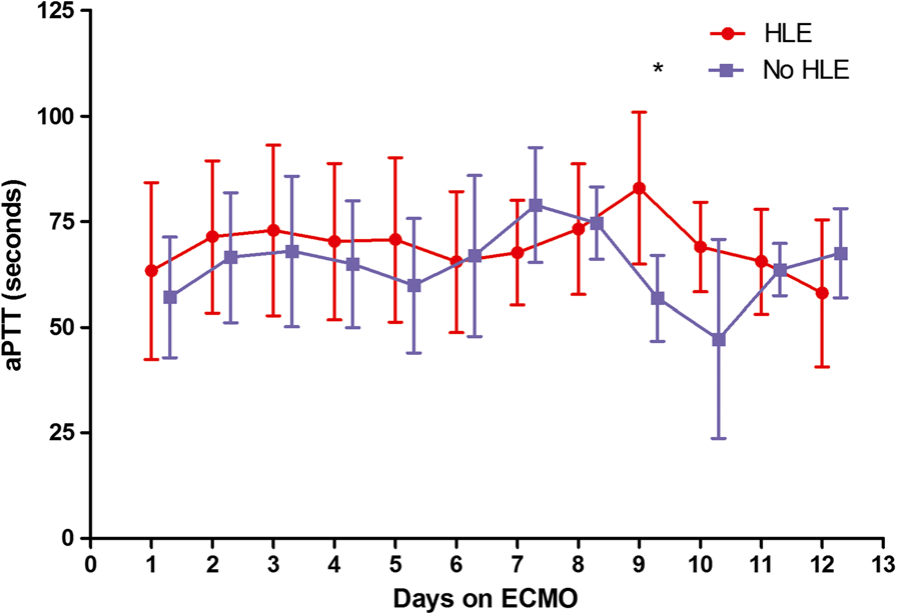
**Acivated partial thromboplastin time (aPTT) in the heparin-like effect (HLE) versus no-HLE groups during the first 12 days on extracorporeal membrane oxygenation (ECMO).** *P <0.05.

**Figure 4:**
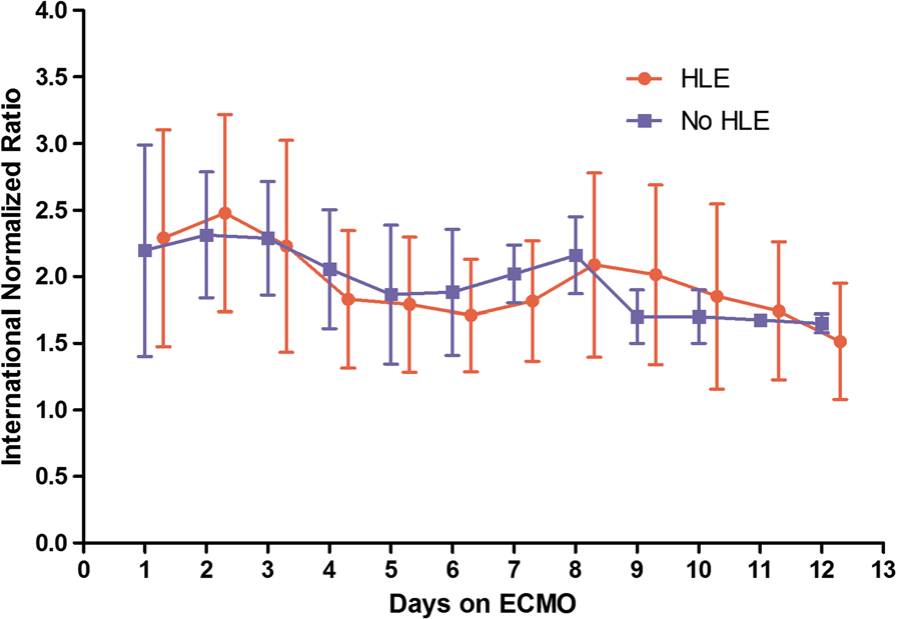
**International normalized ratio (INR) in the heparin-like effect (HLE) versus no-HLE groups during the first 12 days on extracorporeal membrane oxygenation (ECMO)**.

The patterns of aPTT and INR during ECMO are reported in Figures 3 and 4, respectively. The aPTT was longer in the HLE patients during the first 5 days on ECMO, although not significantly. On day 9 the difference became significant (*P* = 0.023). No differences were observed with respect to the INR.

Transfusion data are reported in Table 2. We did not observe significant differences between patients with or without HLE in the general patient population or in the subgroups of pediatric and adult patients.

**Table 2:** **Transfusional needs during ECMO in the patient population**

**Total patient population (n = 41)**	**HLE**	**No HLE**	***P*** **-value**
**Blood product**			
Number of patients	23	18	
Packed red cells, mL/kg/day, mean (SD)	25 (28)	27 (18)	0.783
Fresh frozen plasm, mL/kg/day, mean (SD)	8.9 (14)	11.8 (16)	0.560
Platelet concentrate, mL/kg/day, mean (SD)	3.5 (5.7)	3.9 (4.1)	0.810
**Adult patients (n = 19)**			
**Blood product**			
Number of patients	11	8	
Packed red cells, mL/kg/day, mean (SD)	8.6 (5.6)	12.2 (2.8)	0.134
Fresh frozen plasma, mL/kg/day, mean (SD)	2.2 (2.7)	1.5 (1.9)	0.541
Platelet concentrates, mL/kg/day, mean (SD))	1.7 (1.6)	2.3 (2.7)	0.572
**Pediatric patients (n = 22)**			
**Blood product**			
Number of patients	12	10	
Packed red cells, mL/kg/day, mean (SD)	38.7 (32)	38.6 (16)	0.995
Fresh frozen plasma, mL/kg/day, mean (SD)	14.4 (18)	19.9 (18)	0.494
Platelet concentrates, mL/kg/day, mean (SD)	5.1 (7.3)	5.2 (4.8)	0.979

ECMO: extracorporeal membrane oxygenation; HLE: heparin-like effect.

## Discussion

Our study demonstrates that despite the total absence of UFH during the ECMO period, ECMO patients may exhibit different R-times at the TEG performed with or without heparinase, therefore simulating the presence of circulating heparin. This effect becomes progressively more evident from the ECMO initiation through the fifth day on ECMO, subsequently declining and disappearing after 10 days on ECMO. We could not identify any predictor of the HLE, and the longer ECMO duration observed in patients with HLE is probably simply the expression that the longer the period of observation, the higher is the probability to detect the HLE. It is important to note that HLE has a higher incidence in patients who developed sepsis during the ECMO treatment.

There are different possible explanations for the HLE phenomenon during ECMO. A first possibility is that the difference in R-times with or without heparinase may be ascribed to the well-known inter-examination variations of the TEG technology. TEG has a relatively high coefficient of variation [7]; however, this is usually no greater than 10%. In our series, the mean difference between R-times with or without heparinase exceeded 30% in the first 6 days on ECMO and was almost 100% on day 4 and 5 on ECMO. Therefore, the difference that we observed was largely greater than could be expected for the inter-examination TEG coefficient of variation, and cannot be attributed to this effect.

The most common cause of differences in R-times with or without heparinase is the presence of residual circulating heparin that is not completely antagonized by protamine. In our series, full protaminization was achieved once the ECMO was established, and immediately after ECMO placement, on the first TEG there were significant differences in R-times with or without heparinase. It is therefore unlikely after many days from the last dose of intraoperative heparin that residual effects of this drug will be noticed. However, to rule out this possibility, we have retrospectively analyzed a control group of 12 patients scrutinized with TEG on the first day after surgery due to ongoing microvascular bleeding. Our results clearly demonstrated that no significant differences in R-times were detectable only 24 h after surgery.

A third interpretation can be applied to the cases where a heparin-coated circuit was used. It cannot be excluded that variable doses of heparin may leave the surface of the circuit/oxygenator and enter the systemic circulation while on ECMO. However, we observed the phenomenon in patients treated with phosphorylcholine coatings as well as in patients treated with heparin coatings, and we therefore exclude this interpretation.

Our interpretation is that ECMO patients experience an HLE as observed in other clinical conditions characterized by endothelial disturbance. The endothelial glycocalyx contains the glycosaminoglycans (GAGs) heparan sulfate, chondroitin sulfate and hyaluronan [8]. These substances act like heparinoids, and are scavenged by heparinase [8]. Their release into the systemic circulation determines effects that are similar to those obtained by exogenous UFH. Additionally, GAGs are produced by the mast cells during sepsis or systemic inflammatory reaction syndrome (SIRS) [9].

HLE has been previously demonstrated in three clinical settings: acute liver failure during liver disease and liver transplantation [10,11], sepsis/SIRS [12], and cancer [13]. The mechanisms leading to the HLE are multifactorial, and include (i) failure of the liver to clear the circulating GAGs during acute liver failure [14], (ii) neutrophil-mediated injury of the hepatocytes that can release heparin sulphate [15], and (iii) the direct release of GAGs from the endothelial surface and the mast cells during sepsis/SIRS [15].

The occurrence of an HLE during ECMO may have a multifactorial genesis. During ECMO, the chronic contact of blood with foreign surfaces triggers contact-phase activation, thrombin generation, and activation of the inflammatory system, leading to SIRS. The increased release of cytokines during the SIRS determines an endothelial injury. Additionally, due to the presence of a large number of cannulas and venous catheters, ECMO patients are particularly susceptible to bloodstream infections and sepsis. Sepsis is per se a determinant of cytokine release and direct release of heparinoids from the mast cells [15]. Finally, it cannot be excluded that poor liver perfusion during ECMO may trigger patterns of subacute liver failure. However, in our series we did not detect any association between the peak bilirubin value during ECMO and the peak delta R-time with or without heparinase, and we therefore tend to exclude this last mechanism.

As a matter of fact, in our series the HLE is not associated with a significantly worse outcome in terms of survival (39% in patients with HLE versus 55% in patients without HLE); however, we must admit that this could simply be due to the relatively limited sample size. Actually, the significantly higher rate of septic patients in the HLE group suggests that HLE could be the expression of a potentially severe clinical condition. In about 30% of the patients with HLE, sepsis was probably the leading cause; in the remaining 70%, HLE is likely to be the expression of a natural inflammatory reaction to the blood exposure to foreign surfaces rather than a marker of severe complications.

HLE in ECMO patients has never been reported before. The main reason for this is that the great majority of ECMO patients undergo anticoagulation with UFH. Therefore, no possibility exists to separate the effects of endogenous heparinoids and exogeneous UFH. Our model based on bivalirudin as the sole anticoagulant provided us with the possibility to observe and measure this phenomenon.

There are some limitations in our study. The retrospective nature could determine a risk of bias and of exclusion of variables that may be associated with the HLE phenomenon. Additionally, the relatively low number of patients does not allow multivariable analyses. We must consider the heterogeneity of our patient population, composed of newborn babies, infants, children, and adult patients. Although age was not associated with a different incidence of HLE, we cannot exclude that the well-known differences in the coagulation pattern observed in newborn babies and infants versus adult patients may play a role in the determinism and severity of the HLE.

This is a single-center study, and our results need to be confirmed in larger series of patients, and in patients in whom the ECMO was applied in a non-postcardiotomy setting. Additionally, other techniques for diagnosing an HLE during ECMO (that is, direct measurement of heparin concentration in bivalirudin-treated patients) could confirm our findings.

## Conclusions

The potential therapeutic implications of our findings are still to be defined. We cannot exclude that HLE in patients undergoing anticoagulation with UFH on ECMO may determine, contrary to our study based on bivalirudin anticoagulation, an increased risk of bleeding, especially in the early days on ECMO. Potential interventions include a decrease of the UFH infusion dose, whereas the use of protamine should probably be limited to patients with severe, life-threatening bleeding. Protamine has been successfully used for reversing HLE during liver transplantation [16,17], but of course its use may trigger thromboembolic events in the ECMO setting. In our series, HLE was more common in patients with septic conditions. In this respect, additional studies are certainly needed to confirm HLE as a potential marker of systemic infection.

In conclusion, we think that physicians managing ECMO patients should be aware that a HLE may be frequently found, especially in the first week of ECMO. Detection of the HLE could trigger adequate interventions in terms of UFH dose changes.

## Key messages

- During ECMO without heparinization, the patients experience an HLE in about 50% of cases
- The HLE is evident during the first week on ECMO, and decreases with time after the seventh day on ECMO
- The HLE is not due to liver failure or heparin coating of the ECMO system
- There is an association between the HLE and the onset of sepsis, with a sepsis rate during ECMO of 30% in patients with an HLE, and of 6% in those without this phenomenon.

## Abbreviations

ACT: activated clotting time
aPTT: activated partial thromboplastin time
ECMO: extracorporeal membrane oxygenation
GAGs: glycosaminoglycans
HLE: heparin-like effect
INR: international normalized ratio
R-time: reaction time
SIRS: systemic inflammatory reaction syndrome
TEG: thromboelstography
UHF: unfractionated heparin

## References

[1] Oliver WC (2009). Anticoagulation and coagulation management for ECMO. Semin Cardiothorac Vasc Anesth.

[2] Pollak U, Yacobobich J, Tamary H, Dagan O, Manor-Shulman O (2011). Heparin-induced thrombocytopenia and extracorporeal membrane oxygenation: a case report and review of the literature. J Extra Corpor Technol.

[3] Koster A, Weng Y, Böttcher W, Gromann T, Kuppe H, Hetzer R (2007). Successful use of bivalirudin as anticoagulant for ECMO in a patient with acute HIT. Ann Thorac Surg.

[4] Pieri M, Agracheva N, Bonaveglio E, Greco T, De Bonis M, Covello RD, Zangrillo A, Pappalardo F (2013). Bivalirudin versus heparin as an anticoagulant during extracorporeal membrane oxygenation: a case-control study. J Cardiothorac Vasc Anesth.

[5] Ranucci M (2012). Bivalirudin and post-cardiotomy ECMO: a word of caution. Crit Care.

[6] Ranucci M, Ballotta A, Kandil H, Isgrò G, Carlucci C, Baryshnikova E, Pistuddi V (2011). Bivalirudin-based versus conventional heparin anticoagulation for postcardiotomy extracorporeal membrane oxygenation. Crit Care.

[7] Chen A, Teruya J (2009). Global hemostasis testing thromboelastography: old technology, new applications. Clin Lab Med.

[8] Gao L, Lipowsky HH (2010). Composition of the endothelial glycocalyx and its relation to its thickness and diffusion of small solutes. Microvasc Res.

[9] Koksal M (1953). Extraction of a heparin-like substance from mast cell granules in mouse connective tissue. Nature.

[10] Senzolo M, Agarwal S, Zappoli P, Vibhakorn S, Mallett S, Burroughs AK (2009). Heparin-like effect contributes to the coagulopathy in patients with acute liver failure undergoing liver transplantation. Liver Int.

[11] Senzolo M, Cholongitas E, Thalheimer U, Riddell A, Agarwal S, Mallett S, Ferronato C, Burroughs AK (2009). Heparin-like effect in liver disease and liver transplantation. Clin Liver Dis.

[12] Bulanov AI, Iatskov KV, Shulutko EM, Glukhova TE, Andreĭchenko SA (2012). Endogenous heparin-like syndrome: analysis of clinical observations. Anesteziol Reanimatol.

[13] Fahl KN, Poon SA, Badani KK, Benson MC (2009). Paraneoplastic production of heparin-like anticoagulant in a patient with metastatic transitional cell carcinoma. Can Urol Assoc J.

[14] McKee RF, Hodson S, Dawes J, Garden OJ, Carter DC (1992). Plasma concentrations of endogenous heparinoids in portal hypertension. Gut.

[15] Dhainaut JF, Marin N, Mignon A, Vinsonneau C (2001). Hepatic response to sepsis: interaction between coagulation and inflammatory processes. Crit Care Med.

[16] Pivalizza EG, Abramson DC, King FS (1998). Thromboelastography with heparinase in orthotopic liver transplantation. J Cardiothorac Vasc Anesth.

[17] Bayly PJ, Thick M (1994). Reversal of post-reperfusion coagulopathy by protamine sulphate in orthotopic liver transplantation. Br J Anaesth.

